# Development of ketobenzothiazole-based peptidomimetic TMPRSS13 inhibitors with low nanomolar potency

**DOI:** 10.1080/14756366.2025.2466841

**Published:** 2025-02-20

**Authors:** Alexandre Joushomme, Antoine Désilets, William Champagne, Malihe Hassanzadeh, Gabriel Lemieux, Alice Gravel-Trudeau, Matthieu Lepage, Sabrina Lafrenière, Ulrike Froehlich, Karin List, Pierre-Luc Boudreault, Richard Leduc

**Affiliations:** a Department of Pharmacology-Physiology, Faculty of Medicine and Health Sciences, Université de Sherbrooke, Sherbrooke, Canada; b Department of Pharmacology, Wayne State University, Detroit, Michigan, USA

**Keywords:** Peptidomimetic, TMPRSS13, protease inhibitor, compound screening, SARS-CoV-2

## Abstract

TMPRSS13, a member of the Type II Transmembrane Serine Proteases (TTSP) family, is involved in cancer progression and in respiratory virus cell entry. To date, no inhibitors have been specifically developed for this protease. In this study, a chemical library of 65 ketobenzothiazole-based peptidomimetic molecules was screened against a proteolytically active form of recombinant TMPRSS13 to identify novel inhibitors. Following an initial round of screening, subsequent synthesis of additional derivatives supported by molecular modelling revealed important molecular determinants involved in TMPRSS13 inhibition. One inhibitor, N-0430, achieved low nanomolar affinity towards TMPRSS13 activity in a cellular context. Using a SARS-CoV-2 pseudovirus cell entry model, we further demonstrated the ability of N-0430 to block TMPRSS13-dependent entry of the pseudovirus. The identified peptidomimetic inhibitors and the molecular insights into their potency gained from this study will aid in the development of specific TMPRSS13 inhibitors.

## Introduction

Proteolysis is a finely regulated physiological process, and protease malfunction can lead to a wide range of pathologies^1^. This deregulation of one particular family of proteolytic enzymes, the Type II Transmembrane Serine Proteases (TTSP) family, is associated with a variety of conditions, such as digestive malfunctions, iron overload, cancer, and skin disorders^2^^,^^3^. TTSPs are composed of an N-terminal cytoplasmic tail followed by a transmembrane domain, an intermediate region containing different ancillary domains, and a C-terminal extracellular serine protease domain exposed at the cell surface^4–6^. They are synthesised as single-chain inactive zymogens, requiring proteolytic activation at specific arginine or lysine residues preceding the catalytic domain^5^. Following activation, the catalytic domain remains bound to ancillary domains via a disulphide bridge^6^. Moreover, cell-surface shedding of TTSP ectodomains allows the release of an active form of the enzyme into the pericellular space^7^.

TMPRSS13, a TTSP highly expressed in the skin and to a lesser extent in the proximal digestive tract, contributes to the formation of the epidermal barrier^8^. It is also expressed in the lungs, pancreas, prostate, colon, breast and various tissues of the digestive system^6^^,^^9^^,^^10^. Recent work has revealed its involvement in the progression of both breast and colorectal cancer, where TMPRSS13 silencing augments apoptosis, reduces cell invasion and increases sensitivity to chemotherapeutic agents^10^^,^^11^. Importantly, TMPRSS13 deficiency was associated with decreased tumour growth *in vivo*^10^. These results position TMPRSS13 as an interesting therapeutic target in cancer treatment, where its inhibition could increase the efficacy of commonly used chemotherapies. Additionally, TMPRSS13 is also known as a viral-priming protease because of its ability to activate type I surface glycoproteins of respiratory viruses through proteolytic cleavage, an essential step for their entry into host cells. Hence, TMPRSS13 cleaves and primes SARS-CoV^12^, MERS-CoV^12^, SARS-CoV-2^13–15^, influenza A virus^16^ and highly pathogenic avian influenza virus (HPAIV) spike or haemagglutinin glycoproteins known to harbour multi-basic cleavage sites^17^. Recently, TMPRSS13 was also identified as playing a key role in the onset of Swine Acute Diarrhoea Syndrome (SADS-CoV)^18^. This virus originates in bats^19^, thus presenting a high risk of transmission between species. The implications of TMPRSS13 in various respiratory viruses posing pandemic threats make this protease an important target to consider in the context of antiviral preparedness.

Currently, TMPRSS13 inhibition has been reported with broad-acting serine protease inhibitors such as aprotinin^6^^,^^17^^,^^20^, benzamidine^6^, Bowman-Birk trypsin inhibitors^6^^,^^17^, camostat^18^, nafamostat^15^ and decanoyl-RVKR-CMK, a proprotein convertase inhibitor containing an irreversible chloromethyl ketone warhead^17^^,^^21^. However, no studies aimed at developing inhibitors directed specifically against TMPRSS13 have been reported. We previously achieved potent inhibition of different TTSPs (matriptase^22^, TMPRSS6^23^ and TMPRSS2^24^) via peptidomimetic compounds harbouring a ketobenzothiazole (kbt) warhead, which are able to form a covalent but reversible bond between its ketone and the serine of the protease’s catalytic triad, thereby acting as a serine trap. The inhibition of viral serine proteases has previously been approved by the FDA for the treatment of hepatitis C virus (HCV) ^25^^,^^26^ with Telaprevir and Boceprevir, two protease inhibitors with a mechanism of inhibition similar to that of the ketobenzothiazole warhead. As therapies targeting host factors were also proven to be successful through FDA approval for HCV and HIV^27–29^, the development of TTSP inhibitors is attractive, as evidenced by the recent surge in their interest as potential therapeutic targets^24^^,^^30–32^. Here, the production of active recombinant TMPRSS13 has allowed the screening of a library of peptidomimetic compounds to identify high-affinity inhibitors. The most promising compound was then optimised through the synthesis of derivatives, and important pharmacophores for TMPRSS13 inhibition were identified. *In cellulo*, a proof-of-concept of the most potent compound as a potential antiviral is also presented. Overall, this study reports the development of first-generation potent TMPRSS13 inhibitors with nanomolar activity both *in vitro* and *in cellulo*, which, following further optimisation, could be exploited in different therapeutic contexts.

## Materials and methods

### Cell culture

Simian kidney Vero C1008 (Vero E6, ATCC, CRL-1587) cells were cultured in Eagle’s Minimum Essential Medium (EMEM) (Wisent, 320–005-CL) supplemented with 10% foetal bovine serum (FBS; 080–150 Wisent) and 1% antibiotic mixture (penicillin streptomycin, Wisent, 450–202-EL). Human 293 T (ATCC, CRL-3216) and HEK293SL (gift from Stephane Laporte, McGill, Canada), a 293-cell line subclone selected for enhanced adherence^33^, were maintained in Dulbecco’s modified Eagle’s Medium High Glucose (DMEM) (Wisent, 319–005-CL) supplemented with 10% FBS and 1% antibiotic mixture. Expi293F cells (Thermo Fisher Scientific, A14527) were cultured in Expi293^TM^ expression medium (Thermo Fisher Scientific, A1435101). All the cell lines were cultured in a humidified incubator at 37 °C with 5% CO_2_ in an air atmosphere, except the Expi293F cells, which were cultured with 8% CO_2_.

### DNA constructs

In this study, a variant of TMPRSS13 isoform 1 (GenBank: NM_001077263.3, 567 amino acids), in which one of the five-amino-acid cytoplasmic tandem repeats is missing, was used as the wild type (WT) (GenBank: BC114928.1, 562 amino acids). The TMPRSS13 coding sequence was amplified and inserted into the pcDNA 3.4 TOPO vector using Taq DNA polymerase (New England Biolabs, M0267S) and the pcDNA 3.4 TOPO TA cloning kit (Thermo Fisher Scientific, A14697) to obtain the TMPRSS13-WT construct used in this study. TMPRSS13-AFAAS-6His was then obtained by the substitution of amino acids R558, R560 and K561 and the addition of a 6xHis tag at the C-terminus using the QuikChange Lightning Site-Directed Mutagenesis Kit (Agilent, 210518).

### Expression of TMPRSS13 in HEK293SL cells, activity measurement and immunodetection

HEK293SL cells were transfected with 1 µg of plasmids (empty vector, TMPRSS13-WT or TMPRSS13-AFAAS-6His) via Lipofectamine 3000 (Thermo Fisher Scientific, L3000001) and Opti-MEM (Thermo Fisher Scientific, 31985062) in 6-well plates at a density of 500,000 cells per well. Twenty-four hours after transfection, the cells were washed with PBS (Wisent, 311–425-CL), and HCell-100 media was added (Wisent, 001–035-CL) for 24 h before the samples were collected. A total of 1.5 ml of cell medium was collected before the cells were lysed at 4 °C in a solution containing 1% Triton, 50 mM Tris (pH 7.4), 150 mM NaCl and 5 mM EDTA supplemented with a protease inhibitor cocktail (Millipore Sigma, 11697498001). The lysate was cleared by centrifugation at 15,000 × g and 4 °C for 15 min, and the protein concentration was calculated via a protein assay dye (Bio-Rad Laboratories, 5000006). The cell medium was removed via centrifugation at 15,000 × g and 4 °C for 15 min, and proteolytic activity was measured by monitoring Boc-RVRR-AMC (Bachem, 4018735.0025) cleavage via a BioTek Synergy HTX Multimode Reader (Agilent Technologies). The cell medium was concentrated for Western blotting via Amicon Ultra centrifugal filters (10 K) (Millipore, UFC501096). Equal quantities of proteins from lysates and 30 µl of concentrated media were diluted in Laemmli sample buffer (Bio-Rad Laboratories, 1610747) with 2.5% β-mercaptoethanol and separated on 4–15% SDS-PAGE precast gels (Bio-Rad Laboratories, 4561084). Proteins were transferred to nitrocellulose membranes (iBlot 2 transfer stacks, Thermo Fisher Scientific, IB23001). The primary antibodies used were rabbit anti-TMPRSS13 (1:2,500; Thermo Fisher Scientific, PA5-30935), mouse HRP-linked anti-β-actin (1:1,000; Cell Signalling Technology, 12262S), and rabbit anti-histidine (1:1,000; Cell Signalling Technology, 2365S). When necessary, an HRP-linked goat anti-rabbit secondary antibody (1:1,000; Cell Signalling Technology, 7074) was used. The proteins were visualised via Clarity Western ECL substrate (Bio-Rad Laboratories, 1705061) on an imaging system (Vilber). The membranes were stripped with Restore PLUS Western blot Stripping Buffer (Thermo Fisher Scientific, 46430) for 15 min at room temperature before washing and the addition of new primary antibodies.

### Production of recombinant TMPRSS13 for compound screening

TMPRSS13-AFAAS-6His was expressed via the Expi293 Expression System Kit (Thermo Fisher Scientific, A14635) according to the manufacturer’s instructions and purified via IMAC chromatography. Briefly, Expi293F cells were transfected for 48 h with the TMPRSS13-AFAAS-6His construct, and media containing the shed recombinant protease was injected on a HisTrap Excel column (Cytiva, 17371206) via an ÄKTA Start protein purification system (Cytiva). The bound proteins were then eluted (20 mM sodium phosphate pH 7.4, 500 mM NaCl, and 500 mM imidazole), and the TMPRSS13-containing fractions were dialysed for 16 h at 4 °C in storage buffer (20 mM sodium phosphate pH 7.4, 150 mM NaCl, and 10% glycerol). Proteins were then concentrated via a 10 kDa Amicon Ultra centrifugal filter (Millipore Sigma, UFC8010) and stored at −80 °C.

### Mass spectrometry

The protein preparation was analysed at the Université de Sherbrooke Proteomic Platform to identify the cleavage fragments of TMPRSS13. Proteins were first reduced using 3.24 mM DTT and subsequently alkylated with 13.5 mM iodoacetamide. The digestion buffer, containing 50 mM ammonium bicarbonate (NH4HCO3) and 1 mM CaCl2, was added, and proteins were digested with chymotrypsin (Thermo Scientific, cat# 90056). The resulting digested samples were desalted using C18 tips (Thermo Scientific, cat# 87764), lyophilised, and reconstituted in 1% formic acid before mass spectrometry analysis. Chymotrypsin-digested peptides were separated on a Dionex UltiMate 3000 nano-HPLC system. Ten microliters of sample (containing 2 μg total protein) in 1% formic acid (v/v) were loaded at a flow rate of 4 μL/min onto an Acclaim PepMap100 C18 trap column (0.3 mm inner diameter × 5 mm; Dionex Corp., Sunnyvale, CA). Following trapping and enrichment, peptides were eluted onto a PepMap C18 nanocolumn (75 μm × 50 cm; Dionex Corp.) using a linear gradient of 5–35% solvent B (90% acetonitrile with 0.1% formic acid) over a period of 240 min at a constant flow rate of 200 nL/min. The HPLC system was interfaced with an Orbitrap QExactive mass spectrometer (Thermo Fisher Scientific Inc.) through an EasySpray source. The spray voltage was maintained at 2.0 kV, and the column temperature was controlled at 40 °C. Full-scan MS spectra (m/z range 350–1600) were acquired in the Orbitrap at a resolution of 70,000 with ion accumulation reaching 1,000,000 ions. The 10 most abundant peptide ions detected in the preview scan were subjected to fragmentation by collision-induced dissociation (CID) with a normalised collision energy of 35% and resolution of 17,500, after accumulating 50,000 ions. The maximum fill times were set to 250 ms for full scans and 60 ms for MS/MS scans. Precursor ion charge state screening was enabled, excluding unassigned, singly, septuply, and octuply charged species. The dynamic exclusion feature was limited to 500 entries, with each entry retained for a maximum of 40 s, and a relative mass tolerance window of 10 ppm was applied. The lock mass option was activated for survey scans to enhance mass accuracy. Data acquisition was performed using Xcalibur software, while processing, searching, and quantification were done using MaxQuant software version 1.5.2.8 with the human UniProt database.

### Ketobenzothiazole-based compound library

A complete list of the compounds used in this study is shown in Table S1. The ketobenzothiazole-based compound library used for the initial screening consists of 53 molecules (compounds **1** to **53**) that were synthesised and characterised in previous studies (see Table S1 for references), along with 12 new molecules (compounds **54** to **65)**. Additionally, four derivatives of hit compound **47** were included (compounds **66** to **69**). Following the publication of the preprint of this work on bioRxiv, MedChemExpress (MCE) has listed **66** (N-0430) for sale.

### Synthesis of compounds

**Scheme 1.**
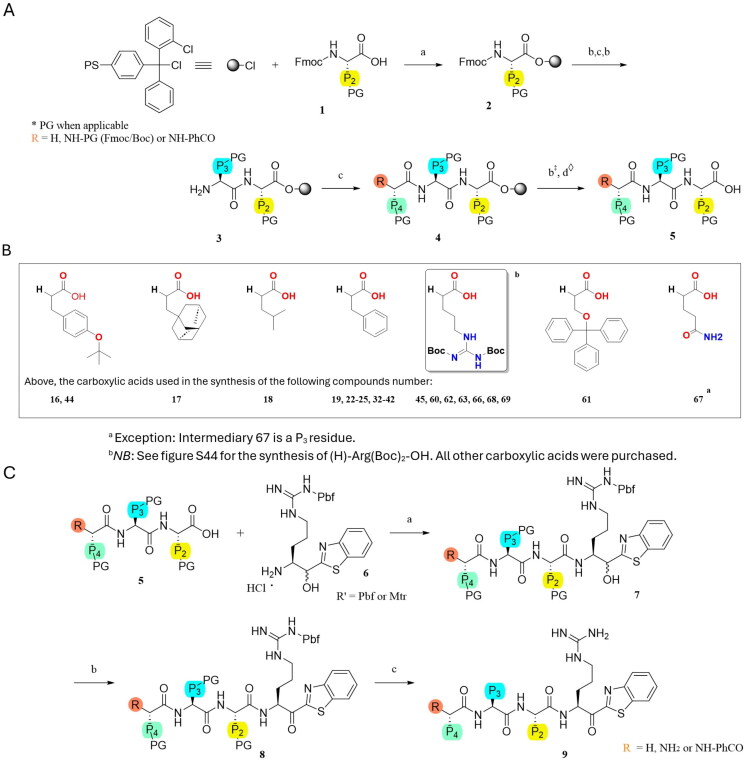
Synthesis. (A) General synthesis of peptide moieties: (a) Fmoc-AA (2 eq.), DIPEA (3 eq.), DCM (b) Piperidine 20%/DMF (c) Fmoc-AA (3 eq.) or **R**-COOH (3 eq.) **(sB)**, HATU (3 eq.), DIPEA (5 eq.), DMF (d) 20% HFIP/DCM, r.t. (‡: When applicable; ◊: Capping group PhCO was added on the N-terminus part using standard peptide coupling conditions c). (B) Carboxylic acids structures coupled at P_4_ for the respective compounds. (C) Warhead coupling to the protected peptides: (a) **7** (1,1), HATU (1,1 eq.), DIPEA (5 eq.), DMF (b) DMP (1,5 eq.), DCM, 0 °C (c) TFA/TIPS/H2O (95:2.5:2.5).

All the compounds were synthesised as described in the general procedure method (Scheme 1). The peptidomimetics were built in two parts, starting with manual solid phase peptide synthesis (SPPS) to obtain the tripeptides (except for compound **67**), which were then isolated. 3 reactions in solution phase enable the obtention of the expected final products. The elongation of the peptide-based inhibitors was carried out as follows (Scheme 1(A), except for compound **65**, see Figure S31). (a) The first Fmoc-protected amino acid [**1**] was loaded on the resin with DIPEA in DCM for 1 h [**2**]. (b) The Fmoc protecting group was removed with 20% piperidine in DMF 2 × 10 min. (c) The next Fmoc-protected amino acid was coupled for 2 h using HATU and DIPEA in DMF. Steps (b) [**3**] and (c) were repeated one more time with amino acid or carboxylic acid (Scheme 1(B)) to obtained desired peptide sequence [**4**]. All Fmoc-protected amino acids and carboxylic acids were purchased from Combi-Blocks, Chem-Impex or Matrix Innovation. (d) The peptide was cleaved from the resin 2 × 45 min using 20% HFIP in DCM at room temperature while keeping the protecting group [**5**]. The warhead [**6**] was added to the tripeptides and the dipeptide (Scheme 1(C)). (a) Amidation of the peptide was carried out with NH_2_-Arg(OH)bt in the presence of HATU and DIPEA in DMF [**7**]. Boc-Arg(OH)bt is commercially available (Piramal Group, Mumbai, India). The Boc protecting group of Boc-Arg(OH)bt was removed using a solution of 4 M HCl in dioxane for 30 min. (b) Oxidation of the secondary alcohol to the ketone was performed using DMP in DCM for 1 h [**8**]. (c) Final deprotection were achieved for 2–3 h using a TFA/TIPS/H2O (95:2.5:2.5) mixture [**9**]. The peptidomimetic compounds were finally purified via preparative high-performance liquid chromatography–mass spectrometry (HPLC-MS), where the major diastereoisomer was isolated (S) when possible, and a purity of >95% was obtained except for compound 61 with a purity of 91.78%. The UPLC chromatograms and MS characterisation data for the newly synthesised molecules and derivatives of hit compound **47** (compound **54** to **69)** are provided in the supplementary data (Figure S4 to S20). The ^1^H and ^13^C NMR spectra (Figure S21 to S30), accurate mass measurements (HRMS, Tables S5 to S10), solid and solution-phase synthesis methods, production intermediates, and isotopic profiles of the most abundant ions (Figure S32 to S44), are also available for the hit derivatives (compounds **66** to **69**).

### In vitro inhibition assay

Recombinant TMPRSS13-AFAAS-6His (this study) and matriptase^34^ were expressed and purified as described. Recombinant human furin, Factor Xa (R&D Systems) and thrombin (MilliporeSigma) were obtained from commercial sources. Assays were performed in a buffer composed of 50 mM HEPES pH 7.4, 1 mM β-mercaptoethanol, 1 mM CaCl2 and 500 μg/ml BSA for furin and 50 mM HEPES pH 7.4, 150 mM NaCl and 0.1% BSA for other proteases. Assays were performed at room temperature using fluorogenic substrates (Boc-RVRR-AMC for furin and TMPRSS13, Boc-QAR-AMC for other proteases). The enzymatic activity was measured via fluorescence (excitation at 360 nm and emission at 460 nm) at room temperature using an HTX Synergy microplate reader (Agilent). *K*_i_s for TMPRSS13 and Factor Xa (FXa) were derived from IC_50_ dose–response curves using the Cheng-Prusoff equation^35^ for reversible competitive inhibitors. For matripase, a plot of the enzyme velocity as a function of the inhibitor concentration was fitted with nonlinear regression analysis via the Morrison *K*_i_ equation^36^ for competitive tight-binding inhibitors.

### In cellulo inhibitory activity and IC_50_ determination

Vero C1008 cells were grown in 12-well plates (200K cells per well) and transfected for 24 h via Lipofectamine 3000 (Life Technologies) with one of the following plasmids: pcDNA3.1 (empty vector, EV), pcDNA3.1-TMPRSS13 WT or pcDNA3.1-TMPRSS13-AFAAS-6His. The cells were then washed 2 times with PBS before HCell-100 medium (Wisent) containing the indicated concentrations of each compound or the control condition (0.01% DMSO) was added. After 24 h of incubation with the compounds, the medium was harvested, and 90 μL was placed in 96-well plate wells with 10 µL of 200 μM Boc-RVRR-AMC. Fluorescence was acquired at room temperature in an FLx800 TBE microplate reader, with excitation at 360 nm and emission at 460 nm. The background was then subtracted by removing the signal recorded with the cells transfected with the empty vector (pcDNA3.1). Proteolytic activity results are represented as a percentage of the DMSO-treated condition. The IC_50_ was calculated via nonlinear regression of the compound concentration relative to the velocity.

### Animals and housing

Adult male Sprague-Dawley rats, weighing between 225 and 300 g (Charles River Laboratories, St. Constant, Canada), were housed in groups of four per double-decker cage on Aspen shavings in a quiet environment. They were maintained under a 12-h light/dark cycle with unrestricted access to food and water. All experimental protocols received approval from the Animal Care Committee of the Université de Sherbrooke, adhering to the Canadian Council on Animal Care guidelines. The procedures also followed the ARRIVE guidelines (Animals in Research: Reporting In Vivo Experiments) and the United States NIH regulations (Ethics approval ID: 2022–3440). To evaluate the stability of compounds in plasma, 6 rats were anaesthetised with 2 L/min oxygen and 5% isoflurane, after which plasma was collected, pooled, and stored frozen until experiments were performed.

### Compound stability

For evaluating plasma stability, rats were anaesthetised using a flow of 2 L/min of oxygen combined with 5% isoflurane. Blood was obtained via cardiac puncture from the left ventricle using a 20 G ½ needle, collected in pre-chilled Vacutainer K2EDTA tubes (367861), and maintained on wet ice. The blood was then centrifuged at 2000 xG for 15 min at 4 °C. Plasma samples were pooled from six rats, aliquoted, and stored at −80 °C. A 96-well plate was prepared with 2.5 μL of the test compound (1 mM aqueous solution) and incubated with 27 μL of pooled plasma (derived from male Sprague − Dawley rats) at 37 °C for different time points: 0, 1, 2, 4, 7, or 24 h (or 0, 5, 15, 30, 60, 90, or 120 min for less stable variants). To terminate the reaction, 140 μL of a 1:1 acetonitrile-ethanol solution containing 0.25 mM N,N-dimethylbenzamide (used as an internal standard) was added. The resulting mixture was transferred onto an Impact Protein Precipitation filter plate (Phenomenex, California), with a 96-well UPLC plate positioned below to collect the filtrates. Both plates were centrifuged at 500 × g for 10 min at 4 °C. The filtrates were diluted with 30 μL of distilled water before analysis using an Acquity UPLC-MS system class H (column Acquity UPLC protein BEH C18 (2.1 mm × 50 mm), 1.7 μm particles, 130 Å pore size). The peptide’s half-life was determined from the degradation profile utilising the exponential one-phase decay function in GraphPad Prism 9. Mass spectra obtained at time points close to the calculated half-life were compared with those at 0 min (plasma inactivated prior to compound addition) to identify cleavage fragments.

For the proteolysis assay, human trypsin (#T6424, Sigma–Aldrich, Saint-Louis, Missouri, USA) was resuspended in 1 mM HCl and 20 mM CaCl2, pH 3, and diluted to a final concentration of 0.1 mg/ml in PBS buffer (pH 7.4, 10 mM PO43-, 150 mM NaCl). The control peptide R8 and the compounds of interest were added to a 96-well plate (37 °C) to a final concentration of 10 µM. Trypsin (0.1 µg/ml) was added to the wells for 0, 0.5, 1, 2, 4 and 6 h. The plate was quenched with AcN/EtOH (1:1) 1% formic acid and 0.25 mM N,N-dimethylbenzamide (IS) and centrifuged at 4 °C. The samples were then analysed via an Acquity UPLC-MS system class H (column Acquity UPLC protein BEH C18 (2.1 mm × 50 mm), 1.7 μm particles with 130 Å pores). The sample half-life was calculated from the degradation curve via the exponential one-phase decay function in GraphPad Prism 9.

### Cytotoxicity

Vero C1008 cells (6,000 cells per sample and 5,000 to −90,000 cells per standard curve) were seeded in 96-well plates. After 24 h, the cells were washed with D-PBS, and EMEM supplemented with 10% FBS and 1% PSG was added. The test compounds (10 µM) were added for 24 h. Cell viability was determined with a CellTiter-Glo 2.0 viability assay kit (Promega, G9242). Cellular viability is expressed relative to that of DMSO-treated cells (vehicle). The luminescence readout was measured via a TriStar LB 942 multimode reader (Berthold Technologies). A standard curve of luminescence readout as a function of the number of cells was obtained by seeding known amounts of cells in the drug-exposed cell plate immediately before adding the reagent. Assays were performed independently at least three times in triplicate. The results were background corrected via negative controls and are presented as the mean cell viability (%) compared with that of vehicle-treated cells ± standard deviation (SD).

### Molecular modelling

Molecular modelling studies were carried out via Molecular Operating Environment (MOE) software, version MOE2022.02. The 3D coordinates of TMPRSS13 and matriptase were obtained from the Protein Data Bank, PDB IDs: 6KD5 and 6N4T, respectively. The models were loaded into the MOE, and polar hydrogens and partial charges were added via the ‘Protonate 3D’ function of the MOE. During the protonation process, a temperature of 300 K, a concentration of 0.1 mol/l salt in the solvent, and a pH of seven were used. Any missing atoms, alternate geometries, or other crystallographic artefacts were fixed via the ‘QuickPrep’ function. The structures were then energy-minimised in the Amber99 force field to achieve a root mean square (RMS) gradient of 0.1 kcal/mol. 3D models were created for all the compounds studied, and their energies were minimised to an RMS gradient of 0.1 kcal/mol via the Amber99 force field. The structures were then protonated, and partial charges were calculated to assign ionisation states and position hydrogens in the macromolecular structure based on its 3D coordinates. Before carrying out the conformational search, a covalent bond was formed between the serine of the catalytic triad and the ketone of the ketobenzothiazole. Compounds N-0388, N-0130, and N-0430 were chosen for molecular modelling studies based on the experimental assay results and were built via the ‘Builder’ tool.

A short molecular dynamics (MD) simulation was conducted on the inhibitor-enzyme complex via MOE and Berendsen equations (BERs). The Berendsen velocity/position scaling methodology was used for system equilibration, as it is insensitive to configuration conditions and suitable for peptide conformations. The guanidine group of the arginine in P1 was constrained by key interactions with Asp at the bottom of the S1 pocket, and a covalent bond was created between the serine of the catalytic triad and the ketone of the warhead. The MD simulations were performed via the AMBER99 force field. The system was neutralised with Na^+^ and Cl^-^ ions at a concentration of 0.1 mol/l, and a Born water model was used for the solvation box. The enzyme-inhibitor solvated complex was energetically minimised for 10 ps. The system was then equilibrated to constant temperature (NVT) and pressure (NPT) conditions at 310 K and 1 atm for 100 ps each. The production step was set to 1 ns at 310 K and 101.325 kPa. Implicit water was used, and the time step was set to 2 fs. The trajectory with the lowest potential energy values was chosen for further analysis. The interactions, geometry, and orientation of all the molecules in the binding cavity were analysed and visualised via MOE. The ‘Align/Superpose’ function in MOE was used to align and superpose the complexes and sequences.

### Virus-like particle (VLP) production and inhibition assay

HEK293T/17 cells were seeded at 6.10^6^ cells per T75 flask for 24 h and were transfected with Lipofectamine 3000 following the manufacturer’s instructions. For transfection, 8 μg of the reporter gene (luciferase-ZsGreen, NR-52516), 6 μg of the helper plasmid (psPAX2, Addgene #12260), and 6 μg of the SARS-CoV-2 spike protein of the delta variant were used. VLPs deprived of surface glycoproteins were produced via the transfection of 6 μg of empty vector (pcDNA3.1) and were used as a negative control. VLP preparations were then filtered through a 0.45 μm filter, aliquoted and stored at −80 °C. The expression of pseudotyped particle components (p24 and S glycoprotein) was validated by immunoblotting. VLPs were normalised by the density of p24 immunoblots using Bio1D software (Vilber Lourmat).

A total of 2 x 10^4^ Vero E6 cells were seeded in 96-well white culture plates and incubated for 24 h. The cells were transfected as described above with 40 ng of TMPRSS13 plasmid or an empty vector (pcDNA3.1) and incubated for 24 h at 37 °C. A 3 h pre-treatment was performed with 10 µM of the test compounds. A normalised preparation of VLPs (δ variant or mock) containing 10 µM of the compounds was added to the wells, and the cells were incubated for 72 h at 37 °C. For measurement, cell culture plates are removed from the incubator and equilibrated to room temperature. A volume of ONE-Glo-EX reagent luciferase assay (Promega, E8130), equivalent to the volume of culture medium, is added to each well, followed by incubation on an orbital shaker at 500 rpm for 5 min. Total luminescence was measured with a Berthold TriStar 2 LB 942 microplate reader.

### Statistical analyses

The data were obtained from at least three different experiments (means ± SDs). Curves, diagrams, and statistical analysis were performed via GraphPad Prism version 10.0.0 for Windows, GraphPad Software (Boston, Massachusetts USA; www.graphpad.com). The size of the error bars indicates the SD within the dataset. All our results are the average of at least 3 independent experiments.

## Results

### Production of a recombinant proteolytically active TMPRSS13 form

**Figure 1.**
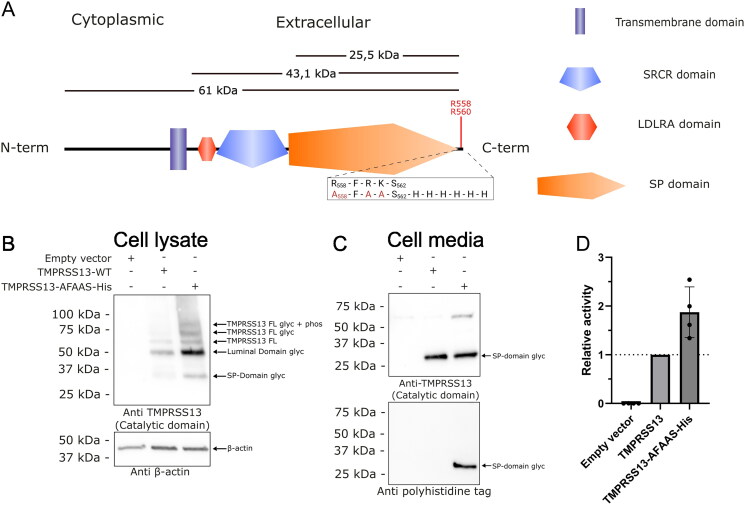
Schematic representation of TMPRSS13 and expression of active recombinant TMPRSS13 forms in HEK293SL cells. (A) Representation of TMPRSS13. Depicted are the different domains of TMPRSS13, the theoretical molecular weight of specific segments, and a close-up view of the his-tagged mutated sequence. In red are noted the amino acids which are subject to spontaneous cleavage following expression and enrichment in the extracellular media (spectra in supplementary data 1). The theoretical weights of 25.5 kDa for the catalytic domain, 43.1 kDa for the extracellular part and 61 kDa for the total protein are given as an indication of what the weights would be without glycosylation. (B) HEK293SL cells were transfected with pcDNA3.1 (empty vector), the full length TMPRSS13 (TMPRSS13-WT) and the his-tagged TMPRSS13 (TMPRSS13-AFAAS-His). Proteins extracted from cell lysates were separated using 15% SDS-PAGE gels under reducing conditions. The separated proteins were then analysed by Western blotting. Proteins were detected with the rabbit C-terminal TMPRSS13 antibody (PA5-30935), targeting the catalytic region of TMPRSS13, and the mouse anti-β-actin antibody (12262S). (C) HEK293SL cells were transfected with an empty vector, the full length TMPRSS13 (TMPRSS13 WT) and the his-tagged TMPRSS13 (TMPRSS13-AFAAS-His). Media concentrates were separated by SDS-PAGE under reducing conditions using 15% gels and then analysed through Western blotting. Proteins were detected with the rabbit C-terminal TMPRSS13 antibody (PA5-30935), targeting the catalytic region of TMPRSS13, or the rabbit anti-His antibody (2365S). In these western blots, FL means Full length, Glyc means Glycosylated, Phos means phosphorylated, and SP is the serine protease domain. (D) Media samples from transfected HEK193SL cells were assessed for TMPRSS13 enzymatic activity using the Boc-RVRR-AMC substrate (100 µM). TMPRSS13 cleaves after an R-R site, releasing the AMC fluorophore. Fluorescence was measured every minute for 60 min. Results were normalised to WT TMPRSS13 specific activity.

To identify TMPRSS13 inhibitors, we first expressed and purified a soluble, active recombinant form of this protein. An initial TMPRSS13 construct containing a fused C-terminal His-Tag (schematic representation depicted in Figure 1(A)) was designed for expression and purification in mammalian Expi293 cells, a commonly used cell line for the production of recombinant proteins^37^, with the intent to harvest and purify the spontaneously occurring shed form of TMPRSS13^7^. However, since TMPRSS13 is known to cleave at arginine or lysine residues^5^^,^^38^, C-terminal cleavages and potential loss of the His-tag were expected to arise during overexpression because of the presence of an Arg-Phe-Arg-Lys-Ser motif located at amino acids 558–562 (Figure 1(A)). To monitor recombinant protease integrity following overexpression in mammalian cells, mass spectrometry analysis after benzamidine purification was performed on the media of Expi293 cells transfected with the TMPRSS13-WT construct. This led to the identification of two spontaneous cleavage sites (R558 and R560) in the last five amino acids of the C-terminal region (Figure 1(A), residues in red, mass spectra available in Figure S1). Near these residues, another basic amino acid (K561) could also potentially be susceptible to cleavage by TMPRSS13. Thus, to prevent the loss of the His-tag, we designed a construct containing protease- resistant residues at these positions (R_558_-F-R-K-S_562_ → A_558_-F-A-A-S_562_).

To assess the expression patterns of the TMPRSS13-AFAAS-6His constructs, HEK293SL cells were transfected, and both the cell lysates and media were analysed via Western blotting (Figure 1(B,C)). Consistent with previous reports^39^, cell lysate analysis via an antibody targeting the protease catalytic domain revealed different TMPRSS13 entities corresponding to a full-length glycosylated and phosphorylated form (∼80 kDa), a glycosylated form (∼70 kDa), a full-length unglycosylated form (∼61 kDa), a cleaved luminal form (∼50 kDa), and, finally, the active glycosylated protease domain (∼30 kDa) (Figure 1(B)). Notably, although the expression pattern was identical for both constructs, the expression levels were greater with the AFAAS-6His construct. Shedding of the TMPRSS13 catalytic domain was detected for both constructs in the cell media via an antibody directed against TMPRSS13 (Figure 1(C), upper panel) and was also observed for the AFAAS-6His construct via an anti-His-Tag antibody (Figure 1(C), lower panel).

Next, to determine whether AFAAS-6His modifications had an impact on TMPRSS13 catalytic activity, cleavage of the Boc-RVRR-AMC fluorogenic substrate by WT or TMPRSS13-AFAAS-6HisTMPRSS13 was monitored in the cell culture media of transfected HEK293 cells (Figure 1(D)). The media of cells transfected with the AFAAS-6His construct did not show a loss of activity but exhibited an increase in specific activity compared with that of the WT enzyme. These results confirm that the TMPRSS13-AFAAS-6His construct is suitable for use as a tool for recombinant protein production of soluble, active proteases, with a similar expression pattern to that of the WT and robust catalytic activity associated with the shed form.

### Identification of potent TMPRSS13 inhibitors

**Figure 2.**
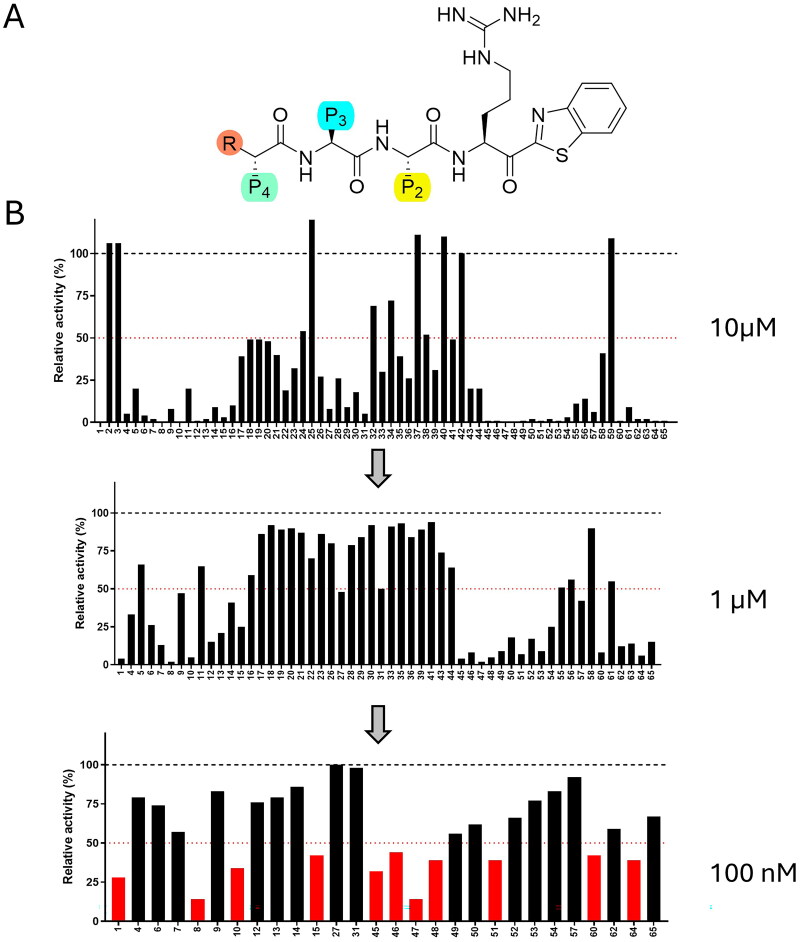
Screening of a 65-compound ketobenzothiazole-based library against TMPRSS13. (A) Scaffold of ketobenzothiazole-based inhibitors. P1 is an Arginine, P2, P3, and P4 were modified with different amino acids. In C-terminal position, a ketobenzothiazole, allows trapping of serine of the catalytic pocket. In N-terminal position, R in orange can be NH_2_, H or NH-PhCO. (B) Compounds that were active at 10 µM were screened at 1 µM then at 100 nM against recombinant TMPRSS13. Compounds affecting > 50% activity were selected for further characterisation.

To identify novel TMPRSS13 inhibitors and important molecular determinants for the inhibition of this protease, we screened a library of 65 peptidomimetic compounds containing various natural and nonnatural amino acids as well as a ketobenzothiazole warhead (see Figure 2(A)) for the general scaffold and Table S1 for the detailed list of all the compounds used in this study) using the TMPRSS13-AFAAS-6His recombinant protein purified from the media of Expi293-transfected cells. Of those 65 molecules, 54 reduced TMPRSS13 activity by more than 50% at a concentration of 10 µM, 30 at 1 µM, and 11 at 100 nM (Figure 2(B)). The ten peptidomimetic compounds that most significantly reduced the relative activity of TMPRSS13 were selected as hit molecules for further characterisation, and for each molecule, a new N-XXXX nomenclature was assigned (Table 1). Interestingly, all hits contained an arginine at the P4 position, and nine of them contained a glutamine at the P3 position, even though only 38% and 40% of the library presented those residues at this position. These findings suggest that a tetrapeptidic scaffold consisting of a P4 Arg and a P3 Gln is preferred to achieve potent inhibition of TMPRSS13.

**Table 1.** *K*_i_ parameters for hits compounds of the original screen.

Screening number	Compounds	Sequence						K_i_ TMPRSS13 (nM)	
		R	P4	P3	P2	P1	Warhead		
1	N-0100	NH_2_	R	Q	A	R	Kbt	31.4	± 9.1
8	N-0107	NH_2_	R	Q	P	R	Kbt	14.7	± 2.6
10	N-0108	NH_2_	R	Q	Y	R	Kbt	22.3	± 6.9
15	N-0117	NH_2_	R	Q	L	R	Kbt	119.9	± 41.9
45	N-0678	H	R	Q	Cha	R	Kbt	30.0	± 10.4
47	N-0159	NH_2_	R	Q	hF	R	Kbt	10.3	± 1.7
48	N-0136	NH_2_	R	Q	F(4F)	R	Kbt	17.7	± 8.3
51	N-0179	NH_2_	hR	Q	A	R	Kbt	24.8	± 5.1
60	N-0439	H	R	A	F	R	Kbt	35.9	± 4.5
64	N-0182	PhCO	R	Q	A	R	Kbt	28.5	± 4.3

*K*_i_ were determined using dose-response curve and the Cheng-Prussof equation for competitive inhibitors.

Next, the *in vitro* inhibition constant (*K*_i_) of these compounds towards TMPRSS13 was determined (Table 1). All selected compounds were confirmed to be potent TMPRSS13 inhibitors in the nanomolar range, and notably, the best compound from this selection, N-0159 (RQhFR-kbt), presented a *K*_i_ of 10.3 nM. Interestingly the second most potent compound, N-0107 (RQPR-kbt with a *K*_i_ of 14.7 nM, Table 1), contains a Pro at the P2 position. The incorporation of a proline residue at the P2 position introduces a rigid, cyclic structure that can significantly impact the binding affinity and specificity of inhibitors targeting TTSPs^40–43^. For instance, the inclusion of proline at the P2 position has been associated with increased TMPRSS2 affinity but also reduced selectivity for other proteases such as factor Xa and thrombin^40^. Exploring the impact of P2-constrained analogs on TMPRSS13 affinity and selectivity is therefore worth investigating. However, given that three of the most effective compounds, including N-0159 (the most potent inhibitor identified), contained phenylalanine or a Phe-derivative at this position, we selected N-0159 as a basis for further optimisation in this study. Derivatives of N-0159 were subsequently synthesised to investigate the contribution of each pharmacophore to TMPRSS13 inhibition (Table 2). We previously demonstrated that modification of the N-terminal amine, such as its deamination, was essential to achieve compound stability in biological contexts^22^. Thus, the N-0159 secondary N-terminal amine was removed, yielding Compound N-0430 ((H)RQhFR-kbt). This led to an inhibitor with a twofold increase in potency at 5.3 nM. At the P2 position, the replacement of the unnatural homo-Phe (N-0430) with the proteogenic amino acid Phe (N-0130) led to a slight decrease in activity (*K*_i_ = 25 nM for N-0130). Finally, the removal of Arg at the P4 position in N-0388 resulted in a drastic reduction in TMPRSS13 inhibition, yielding a compound with a *K*_i_ in the µM range (1.4 µM). Having identified N-0430 as our lead compound, we synthesised N-0430(OH), harbouring a hydroxyl instead of a reactive ketone, which prevents the serine trap from being active. This compound was used as a negative control and, as expected, did not inhibit TMPRSS13 at 10 µM^44^. Hence, characterisation of N-0159 derivatives led to the identification of a potent, low-nanomolar TMPRSS13 inhibitor and confirmed that the presence of an Arg at the P4 position in the peptidomimetic is a crucial molecular determinant for its inhibition.

**Table 2.** *K*_i_ parameters for N-0159 derivatives.

Compounds	Sequence						*K*_i_ TMPRSS13 (nM)	
	R	P4	P3	P2	P1	Warhead		
N-0430	H	R	Q	hF	R	Kbt	5.3	± 1.4
N-0130	H	R	Q	F	R	Kbt	24.6	± 7.3
N-0388	H	–	Q	F	R	Kbt	1443	± 1071
N-0430(OH)	H	R	Q	hF	R	(OH)bt	> 10 000	

*K*_i_ were determined using dose-response curve and the Cheng-Prussof equation for competitive inhibitors.

### Insights into the pharmacodynamic proprieties of TMPRSS13 inhibitors

**Figure 3.**
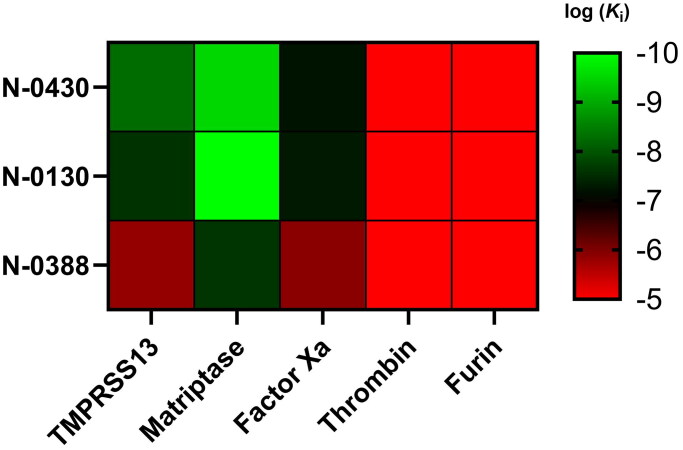
Selectivity heatmap of peptide inhibitors against fives targets. The heatmap displays the selectivity profiles of three compounds (N-0430, N-0130, and N-0388) tested against TMPRSS13, matriptase, Factor Xa, thrombin, and furin. The colour scale on the right ranges from red (log(*K*_i_) = -5) to green (log(*K*_i_) = -10), indicating the logarithmic inhibitory constant (*K*_i_) values for each compound-enzyme pair. Higher log(*K*_i_) values (red) represent weaker inhibition, while lower log(*K*_i_) values (green) indicate stronger inhibition. Data are available in Table S2.

To investigate inhibitor selectivity, Compounds N-0130, N-0430 and N-0388 were tested against a small panel of proteases, including matriptase, another TTSP implicated in cancer and viral entry^32^, as well as potential off-target proteases (FXa, thrombin and furin) (Figure 3, Table S2). N-0430 and N-0130 were found to be subnanomolar inhibitors of matriptase, thus demonstrating better affinity for this protease than TMPRSS13. Removal of the P4 Arg in N-0388 also led to a decrease in affinity, as in TMPRSS13, but the compound remained a potent matriptase inhibitor with a *K*_i_ of 24.2 nM. Compounds N-0430 and N-0130 also achieved nanomolar inhibition of FXa, although to a lesser degree than against TMPRSS13, with *K*_i_ values of 57 and 48 nM, respectively. Similar to TMPRSS13 and matriptase, a loss of potency was detected after removing the P4 Arg in N-0388, with a *K*_i_ of 1.2 µM against FXa. Finally, the three compounds do not inhibit thrombin or are weak inhibitors of both thrombin and furin. These results suggest that this first generation of TMPRSS13 inhibitors exhibits partial selectivity, with reduced potency against other families of serine proteases but high affinities against a member of the same family, matriptase. Interestingly, the introduction of a P4 Arg was essential for achieving nanomolar inhibition of TMPRSS13. Similarly, P4 Arg positively impacted matriptase inhibition, although it was not necessary to achieve nanomolar inhibition of matriptase.

**Figure 4.**
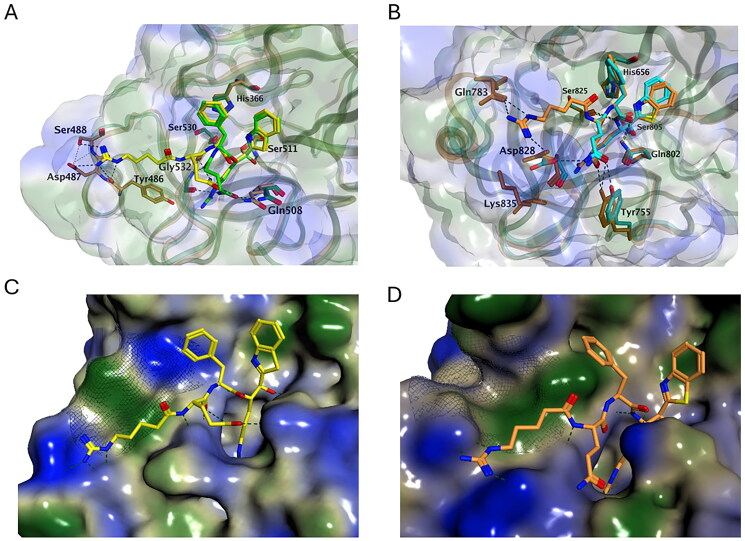
Molecular modelling. (A) Docking of N-0130 ((H)RQFR-Kbt, yellow for compound and dark yellow for ligands) and N-0388 ((H)QFR-Kbt, green for compound and dark green for ligands) to TMPRSS13. (B) Docking of N-0130 ((H)RQFR-Kbt, orange) and N-0388 ((H)QFR-Kbt, cyan) to matriptase. (C) Docking of N-0130 ((H)RQFR-Kbt, yellow) in TMPRSS13. (D) Docking of N-0130 ((H)RQFR-Kbt, orange) in Matriptase. For all panels, hydrophilic part of inhibitor is depicted in blue, hydrophobic in green, and residues involved in interactions are illustrated with dashed lines with each inhibitor are represented in stick form and labelled.

To better understand the impact of the addition of an Arg at the P4 position for TMPRSS13 inhibition, we performed molecular modelling. The catalytic pockets of Compounds N-0130 ((H)RQFR-Kbt) and N-0388 ((H)QFR-Kbt) were first superimposed inside TMPRSS13 (Figure 4(A), 2(D) interaction diagrams available for both compounds in Figure S2). Constant interactions across compounds were observed through the formation of a similar hydrogen bond network between P1 Arg and residues Asp505, Ser506 and Gly534 of the S1 pocket. However, the inclusion of a supplementary Arg in P4 of N-0130 led to several important interactions, each at approximately −9 to −8 kcal/mol (Table S3), with residues Tyr486, Asp487 and Ser488 present in the S4 pocket of TMPRSS13. Optimal binding at the P4 position required a noticeable conformational shift in the N-0130 peptide backbone, which led to a weakened interaction between its P3 Gln from Gln508 compared with N-0388 (-2.4 vs. −4.5 kcal/mol, respectively). A similar but more subtle phenomenon was observed in matriptase (Figure 4(B)), where the addition of a P4 Arg in N-0130 led to an increase in interactions with Gln783. However, the P3 Gln in N-0388 can interact with both residues Asp828 and Tyr755 of matriptase, compensating slightly for the lack of P4, which is consistent with the *in vitro* results. The preference of TMPRSS13 for tetrapeptides is also reflected in the overall shape of its S4 substrate pocket (Figure 4(C)) compared with that of matriptase (Figure 4(D); detailed interaction properties are available in Table S4). Both pockets are similar in length but differ strikingly in their layout. TMPRSS13 has a narrow hydrophobic pocket resulting from Tyr489 and Asp411 hydrogen bond formation, whereas the corresponding amino acids in matriptase (Phe708 and Gln783) do not interact, forming an open groove. Thus, the TMPRSS13 S4 pocket is more restrictive, limiting inhibitor size and flexibility, whereas matriptase is more accommodating.

**Figure 5.**
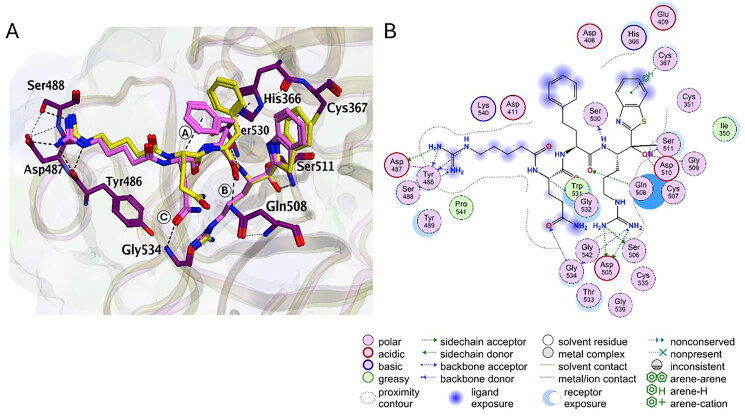
Representation of the interactions between N-0430 and TMPRSS13. Representations are in 3D (A) and 2D (B). N-0430 is shown in light pink stick mode and TMPRSS13 shown in purple stick mode. For comparison, N-0130 is superimposed and represented in yellow stick.

We observed that the presence of P2 hPhe in N-0430 led to an increase in affinity of ~ 5-fold compared with N-0130 containing the natural Phe at this position. To explain this increase in potency, both compounds were docked inside the catalytic pocket of TMPRSS13 (Figure 5, Table S3). The side chain elongation in hPhe not only allows better filling of the binding pocket but also enforces an intramolecular H-arene interaction between the aromatic cycle at the P2 position and the α-carbon of the P3 position (position A), which stabilises its conformation; this allows N-0430 to interact with the carbonyl group in the backbone of its P2 position with Gln508, whereas N-0130 uses its P3 Gln to interact with this residue (position B). Although those two different interactions have similar potencies for both compounds (-2.3 kcal/mol for N-0430 and -2.4 kcal/mol for N-0130), the P2 backbone interaction in N-0430 frees its P3 Gln to achieve an additional H-bond with Gln534 (position C), thus increasing the interaction with this residue (-8.1 kcal/mol for N-0430 and -4.6 kcal/mol for N-0130). Furthermore, the N-0430 optimised conformation in TMPRSS13 also allows for an additional interaction with Asp487, lowering the interaction energy of −5.24 kcal/mol compared with N-0130. These results support the increased affinity of N-0430 for TMPRSS13 compared with N-0130. Overall, these results provide insights into compound selectivity and enabled us to determine the molecular interactions that favour TMPRSS13 inhibition.

### In cellulo potency and stability of TMPRSS13 inhibitors

**Figure 6.**
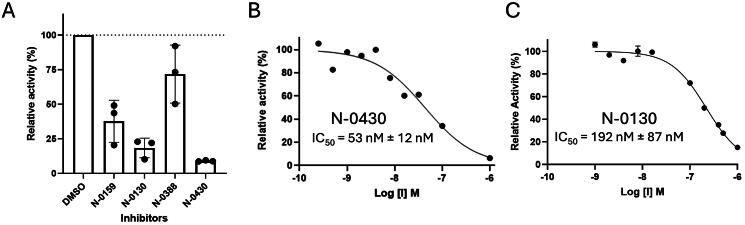
Evaluation of the potency in a cellular context. Vero cells were transfected with WT TMPRSS13, and activity was monitored using Boc-RVRR-AMC as a fluorogenic. (A) Relative activity of TMPRSS13 in the presence of 10 µM of compounds (N-0159, N-0430, N-0130, N-0388 and N-0430-OH) or vehicles (DMSO), displaying relative activity as a percentage of DMSO. (B) Relative activity of TMPRSS13 as a function of N-0430 concentration. (C) Relative activity of TMPRSS13 as a function of N-0130 concentration.

To assess whether the inhibitors were potent in the cellular environment, the effects of the compounds were next screened at 10 µM in WT TMPRSS13-transfected Vero cells, and the media were harvested to measure the activity (Figure 6(A)). The potent inhibition of TMPRSS13 was observed for both the deaminated tetrapeptides N-0130 (18.4% residual activity) and N-0430 (9.0% residual activity), which were more effective than N-0159 (37.7% residual activity) containing the unmodified N-terminal amine. In line with the *in vitro* results, N-0388 presented limited inhibition of TMPRSS13, with residual activities of 72% and 92%, respectively. Next, *in cellulo* TMPRSS13 inhibition by N-0130 and N-0430 was determined through the generation of dose–response curves (IC_50_) via the same assay (Figure 6(B,C)). With an IC_50_ of 53 nM, N-0430 was found to be ~4-fold more potent than N-0130. These results demonstrate that N-0430 is a potent cellular inhibitor of TMPRSS13, which was thus identified as the lead compound of this study.

We next sought to investigate the preliminary pharmacokinetic properties of the selected compounds. To do so, the degradation rates of the peptidomimetics N-0130, N-0159 and N-0430 were determined in the presence of human trypsin and in rat plasma (Table 3). N-0130 presented slightly better stability than the other compounds when incubated with trypsin, but all the molecules achieved a half-life > 1 day. In rat plasma, however, the concentration of Compound N-0159 drastically decreased, with a calculated half-life of 9.0 min, whereas both deaminated compounds were quite stable, with measured half-lives of 3.3 and 1.7 days for N-0130 and N-0430, respectively. As expected, these results demonstrate that the deamination of Compounds N-0130 and N-0430 improved their biological stability. To ensure that these results were not due to cytotoxicity, cellular toxicity tests were carried out with these different compounds, and none were identified as toxic at the highest concentration used (10 µM; Figure S3).

**Table 3.** Stability of peptidomimetics N-0130, N-0159 and N-0430.

Condition	Compounds	Mean half-life ± SD (days)	
Trypsin	N-0130	**4.37**	± 0.71
	N-0159	**1.60**	± 0.40
	N-0430	**1.23**	± 0.31
Plasma	N-0130	**3.27**	± 1.55
	N-0159	**8.97 min**	± 0.15 min
	N-0430	**1.70**	± 0.20

N-0130, N-0159 and N-0430 were put in the presence of proteolytic enzyme (human Trypsin), (final conc. 10 µM) or of rat plasma (male) (final conc. 0.2 mM).

### Effect of N-0430 on the cellular entry of SARS-CoV-2 delta pseudovirus

**Figure 7.**
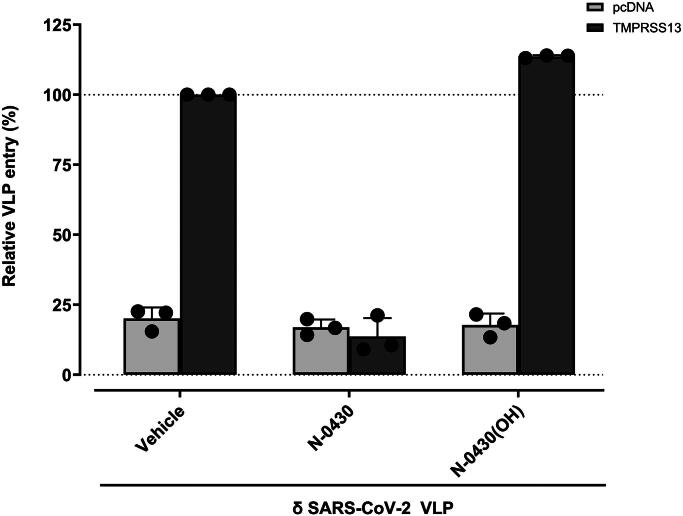
δ SARS-CoV-2 VLPs entry in VERO E6 cells transfected with TMPRSS13 in presence of N-0430, N-0430(OH) and vehicles. VLPs infection in the presence of 10 µM of N-0430 or N-0430(OH) relative to the vehicle-treated condition (DMSO condition). Cells were transfected with TMPRSS13 WT 24 h before incubation with VLP. Compounds were added 3 h before and during the 72 h VLP incubation on cells. The background was subtracted from all particles using a VLP without surface glycoprotein for both experiments. Each experiment was repeated at least three times with vehicle condition, N-0430 and N-0430(OH), with five technical replicates per biological replicate.

TMPRSS13 has been associated with the priming of viruses, including SARS-CoV-2^12,^^13^^,^^17^^,^^18^. To evaluate the potential use of these TMPRSS13 inhibitors, we developed a proof-of-concept assay using virus-like particles (VLPs) pseudotyped with the SARS-CoV-2 delta variant. Vero E6 cells transfected with either an empty plasmid (pcDNA3.1) or TMPRSS13-WT were infected with VLPs in the presence of vehicle (DMSO), N-0430, or N-0430(OH) (Figure 7). TMPRSS13 expression increased VLP entry under vehicle conditions, whereas treatment with 10 µM N-0430 completely abolished TMPRSS13-dependent VLP entry. Conversely, its inactive homolog, N-0430(OH), did not inhibit VLP entry. These results confirmed that N-0430 achieved *in cellulo* inhibition of TMPRSS13 and demonstrated the potential use of this class of compounds for TMPRSS13-dependent pathological processes.

## Discussion

Given the different roles of TMPRSS13 in pathologies such as cancer^10^^,^^11^ and viral entry^13^^,^^17^, selective enzymatic inhibitors could be valuable tools either for gaining new insights into the functions of TMPRSS13 or as a potential therapeutic agent. However, the availability of such inhibitors has been limited, with only broad-spectrum or irreversible inhibitors currently available.

In this study, a shed, soluble and active form of TMPRSS13 enabled the screening of a compound library against this protease. This led to the characterisation of a first-generation set of TMPRSS13 inhibitors and the identification of crucial molecular interactions contributing to their potency, thus representing a significant advancement in the development of selective inhibition of TMPRSS13. From an initial screening of 65 compounds, N-0159 was identified as the most potent. The synthesis of analogs of this compound led to the discovery of N-0430, which exhibited an *in vitro K*_i_ of 5.3 nM towards TMPRSS13. Compared with thrombin and furin, the compounds were more selective for TMPRSS13, for which they showed weak or no inhibition. However, they are potent inhibitors of FXa, a serine protease implicated in the blood coagulation cascade^45^, and matriptase, another TTSP of the same subfamily implicated in cancer as well as in viral entry^5^. This was expected since this class of compounds had previously achieved potent inhibition of these proteases^22^^,^^46^. Thus, depending on the mode of administration, the desired targets and pathological conditions for treatment, further optimisation of these compounds will be necessary to achieve selectivity towards these enzymes.

Compound N-0430 still represents a significant improvement over broad-spectrum serine protease inhibitors, such as aprotinin, benzamidine, or nafamostat, which have been used to inhibit TMPRSS13^6,^^18^. Hence, these inhibitors lack targeted discrimination, inhibiting TTSPs but also serine proteases of other families such as furin and thrombin ^47–49^, which is not the case for N-0430. However, we still observe significant potency against related proteases, similar to what is reported for other ketobenzothiazole-based TTSP inhibitors^23^^,^^44^. Increasing the selectivity for TMPRSS13 would not only reduce the risk of side effects in therapeutic applications but also provide a valuable research tool, enabling more precise investigations on TMPRSS13 function in viral infections and cancer biology.

The developed tetrapeptides N-0130 and N-0430, which harbour a deaminated amino acid at the P4 position, demonstrated increased stability and were proven to be nanomolar *in cellulo* inhibitors of TMPRSS13. The inhibition of TMPRSS13 by N-0430 in a cellular context was further confirmed by its ability to block the entry of pseudotyped SARS-CoV-2 VLPs. These results are consistent with the inhibition of TMPRSS13-dependent SARS-CoV-2 entry or cell fusion by camostat in other cell lines^15^^,^^50^^,^^51^. In addition to betacoronaviruses^13^^,^^52^, TMPRSS13 has also been reported to activate monobasic cleavage sites of influenza A viruses^12^^,^^20^ and, more recently, SADS-CoV-2^18^. However, its role was found to be more pronounced in the activation of highly pathogenic influenza viruses (HPAIVs) of the H5 subtype, characterised by a multibasic cleavage site containing a lysine at the P4 position (KKKR↓G), which are poorly activated by either furin or PC5/6^17^^,^^53^. Although cleavage sites of recent H5N1 HPAIV outbreaks of clade 2.3.4.4b harbour an Arg at the P4 position (RRKR↓G)^54^, suggesting cleavage by furin, sequences prone to TMPRSS13 proteolysis can naturally occur, as they were previously detected in poultry^55^. Thus, selective TMPRSS13 inhibition or its inclusion in a panviral priming protease inhibitor could be of significant interest.

Inhibition of TMPRSS13 proteolytic activity could also be valuable for investigating its role in cancer and developing therapeutics. TMPRSS13 silencing is currently employed to decipher its implication in colorectal and breast cancer^10^^,^^11^. Although the use of siRNA effectively reduces expression, it does not discriminate between the loss of enzymatic activity and the disruption of protein–protein interactions. Therefore, the development of TMPRSS13 inhibitors could provide a supplementary tool to distinguish these functions, thereby advancing our understanding of the role of TMPRSS13 in oncogenesis^10^.

SAR analysis via *in vitro* results and molecular modelling provided insights into key structural elements required to achieve nanomolar potency towards TMPRSS13. This analysis helps us better understand why our lead compound, N-0430, features an hPhe at the P2 position and an Arg at the P4 position. The gain in potency, through elongation of the Phe residue, is proposed to occur via an intramolecular interaction that stabilises the optimal compound conformation and improves pocket occupancy, leading to more potent interactions with the TMPRSS13 residue. Furthermore, the presence of an Arg at the P4 position was confirmed to be critical for TMPRSS13 inhibition, as its removal led to a substantial decrease in compound potency. At this position, the TMPRSS13 catalytic site presented a closed shape, and the presence of amino acids complementary to the positively charged guanidinium group of Arg led to the formation of several crucial potent interactions. This finding correlates with the TMPRSS13 crystal structure, which shows that the TMPRSS13 substrate pocket recognises both Arg and Lys residues at the P4 position^21^.

The docking poses of the tri- or tetrapeptidomimetic compounds were also compared with those of the matriptase binding sites. Similar to TMPRSS13, the removal of Arg at the P4 position led to a decrease in compound potency towards matriptase. This was expected, as this moiety was reported to benefit substrate cleavage or matriptase inhibition^56^^,^^57^. However, it was not necessary to achieve nanomolar inhibition of matriptase, as was the case for TMPRSS13. Modelling studies highlight a more open S4 pocket in matriptase, which could accommodate alternative inhibitor conformations. The identified differences in the overall shape of the S4 pocket between TMPRSS13 and matriptase could be exploited through the use of arginine or lysine analogs and isosteres to achieve selectivity. Recently, the use of a hybrid combinatorial substrate library (HyCoSul) led to the identification of several unnatural P4 residues that can modulate the potency and specificity of matriptase, hepsin and hepatocyte growth factor activator (HGFA)^57^^,^^58^. This finding suggests an opportunity to further increase the affinity of compounds for TMPRSS13 compared with other TTSPs, such as matriptase.

Overall, this study presents the first generation of ketobenzothiazole-based inhibitors designed to target TMPRSS13. Among these, N-0430 was demonstrated to be a potent *in cellulo* inhibitor of this protease. Further optimisation of these compounds will be necessary to achieve selectivity against closely related TTSPs. Moreover, given the potency of N-0107, which contains a proline at the P2 position, constrained analogs at this position, along with a series of macrocycles designed to enhance rigidity, could be the focus of a subsequent study. These compounds could serve as molecular tools to confirm the involvement of TMPRSS13 proteolytic activity in various pathologies, including colorectal and breast cancers. Moreover, TMPRSS13 inhibition could have significant potential for antiviral preparedness, particularly in the development of broad-spectrum antiviral therapies.

## Supplementary Material

Supplementary Material_ Clean.docx

## Data Availability

The authors confirm that the data supporting the findings of this study are available within the article or its supplementary materials.

## Interest statement

P.-L.B. and R.L. are inventors on patent applications (US9365853B2 and US10988505B2) that cover matriptase and other type II transmembrane serine protease inhibitors for treating and preventing viral infections, respiratory disorders, inflammatory disorders, pain disorders, tissue disorders, hyperproliferative disorders, and disorders associated with iron overload. The remaining authors declare that they have no competing interests.
